# New Diagnosis of AIDS Based on* Salmonella enterica *subsp. I (*enterica*) Enteritidis (A) Meningitis in a Previously Immunocompetent Adult in the United States

**DOI:** 10.1155/2017/1051975

**Published:** 2017-08-14

**Authors:** Andrew C. Elton, James Levin, Matthew P. Lazio

**Affiliations:** ^1^Division of Pharmaceutical Sciences, University of Wisconsin-Madison School of Pharmacy, Madison, WI, USA; ^2^Madison Emergency Physicians, Madison, WI, USA; ^3^Division of Infectious Disease, Department of Internal Medicine, SSM Health Dean Medical Group, Madison, WI, USA; ^4^Division of Infectious Disease, Department of Internal Medicine, University of Wisconsin School of Medicine and Public Health, Madison, WI, USA; ^5^Department of Emergency Medicine, SSM Health St. Clare Hospital, Baraboo, WI, USA; ^6^Department of Family Medicine, University of Wisconsin School of Medicine and Public Health, Madison, WI, USA

## Abstract

*Salmonella* meningitis is a rare manifestation of meningitis typically presenting in neonates and the elderly. This infection typically associates with foodborne outbreaks in developing nations and AIDS-endemic regions. We report a case of a 19-year-old male presenting with altered mental status after 3-day absence from work at a Wisconsin tourist area. He was febrile, tachycardic, and tachypneic with a GCS of 8. The patient was intubated and a presumptive diagnosis of meningitis was made. Treatment was initiated with ceftriaxone, vancomycin, acyclovir, dexamethasone, and fluid resuscitation. A lumbar puncture showed cloudy CSF with Gram negative rods. He was admitted to the ICU. CSF culture confirmed* Salmonella enterica *subsp. I (*enterica*) Enteritidis (A). Based on this finding, a 4th-generation HIV antibody/p24 antigen test was sent. When this returned positive, a CD4 count was obtained and showed 3 cells/mm^3^, confirming AIDS. The patient ultimately received 38 days of ceftriaxone, was placed on elvitegravir, cobicistat, emtricitabine, and tenofovir alafenamide (Genvoya) for HIV/AIDS, and was discharged neurologically intact after a 44-day admission.

## 1. Introduction

*Salmonella*, a Gram negative bacillus, with greater than 2500 serovars, is a rare etiologic agent of infection in the United States and other developed areas. Most frequently seen in developing, AIDS-endemic countries, infection may present in a variety of ways including enteritis, sepsis, focal infection, or chronic carriage [1, 2]. Of these,* Salmonella* meningitis is a rare manifestation typically resulting from foodborne infection. In* Salmonella* meningitis, a 37% mortality rate has been reported, and it typically presents in patients under 4 or above 60 years of age [3, 4].

Many reports describe* Salmonella* meningitis in the setting of an immunocompromised state, such as those patients with a previously known diagnosis of HIV [1, 5–7]. In the setting of AIDS, the gastrointestinal tract becomes highly susceptible to opportunistic infections from the diet, allowing for focal infections like meningitis to develop. Worldwide, there is one report of a previously healthy adult in Greece with* Salmonella* meningitis ultimately leading to the diagnosis of AIDS [5]. We present the first case, to our knowledge, of* Salmonella* meningitis leading to a new diagnosis of AIDS in an adult patient in the United States.

## 2. Case Report

A 19-year-old Eastern European seasonal worker in the United States on a J-1 Visa with no documented medical history was brought to a rural Wisconsin emergency department with altered mental status. A welfare check had been initiated because the patient had not reported for his seasonal job at a waterpark in a tourist area for 3 days. The police found the patient in his residence unresponsive and not following commands.

Initial ED vitals showed temperature of 39.8°C (103.7°F), heart rate of 146 beats/min, respiratory rate of 27 breaths/min, blood pressure of 109/53 mmHg, and O_2_ saturation of 99% on room air. Physical exam was remarkable for delirium with GCS of 8 (E2, V2, M4), tachycardia without murmur, tachypnea with clear lung exam, no abdominal guarding, and no signs of external trauma. He was not following commands and not protecting his airway.

He immediately underwent rapid sequence intubation using ketamine for induction and succinylcholine for paralysis. He was sedated after intubation with propofol. Given his presentation and physical exam, a presumptive diagnosis of severe sepsis from meningoencephalitis was made, and ceftriaxone, vancomycin, acyclovir, and dexamethasone and fluid resuscitation were immediately initiated while an exhaustive diagnostic workup was begun.

Initial ED laboratory values returned CBC with WBC 4.5 cells/mm^3^, hemoglobin 7.1 g/dL, hematocrit 23%, platelets 53 × 10^3^/*µ*L, CMP with sodium 145 mmol/L, potassium 3.0 mEq/L, chloride 116 mmol/L, CO_2_ 16 mmol/L, glucose 156 mg/dL, BUN 48 mg/dL, creatinine 1.29 mg/dL, AST 79 IU/L, ALT 17 IU/L, bilirubin 1.4 mg/dL, albumin 2.8 g/dL, and calcium 7.3 mg/dL. Lactate was 1.4 mmol/L, troponin 0.07 ng/mL, CK 659 IU/L, D-dimer 6.81 *μ*g/mL, fibrinogen 517 mg/dL, and TSH 0.67 U/mL. UA showed 3–5 RBCs/hpf, 6–10 WBCs/hpf, 3–5 granular casts, and no bacteria. PT/INR, PTT, and urine drug screen were unremarkable.

CT head and chest X-ray were unremarkable. Lumbar puncture (LP) revealed cloudy fluid with an opening pressure of 29 cm H_2_O. CSF analysis showed 12,301 nucleated cells, 98% of which were polymorphonucleocytes. CSF Gram stain revealed Gram negative rods. The patient was transferred to a tertiary care center with a presumptive diagnosis of Gram negative meningitis and admitted to the medical intensive care unit (MICU).

Two sets of blood cultures grew Gram negative rods in <24 hours. CSF and blood cultures both returned* Salmonella enterica *subsp. I (*enterica*) Enteritidis (A) susceptible to ampicillin, ceftriaxone, ciprofloxacin, and trimethoprim/sulfamethoxazole.

In the MICU, given the unusual presentation of* Salmonella* meningitis, a search for a cause of an immunocompromised state and other opportunistic infections was undertaken. To evaluate this, a 4th-generation HIV-1/HIV-2 antibody and HIV p24 antigen panel was ordered and returned positive. Antibody differentiation confirmed HIV-1 infection. HIV RT-PCR returned 560,000 copies/mL. A CD4 count was obtained showing 3 cells/mm^3^ and CD4% of 0.4%, confirming AIDS. Genotyping and integrase inhibitor resistance assays were negative, confirming a wild-type virus. He was started on elvitegravir, cobicistat, emtricitabine, and tenofovir alafenamide (Genvoya).

Given his new HIV diagnosis, RPR,* Toxoplasma* IgG/IgM, CMV IgG/IgM, CMV DNA PCR, and mycobacterial blood cultures were obtained to rule out accompanying opportunistic infections.* Toxoplasma* IgM, CMV IgM, and CMV DNA PCR all returned negative. Hepatitis screen was negative. QuantiFERON Gold was indeterminate.* Toxoplasma* and CMV IgG results returned positive, confirming past disease. He was started on trimethoprim/sulfamethoxazole and azithromycin for* Pneumocystis jirovecii* pneumonia and* Mycobacterium avium* complex (MAC) prophylaxis, respectively.

By hospital day 2, his blood cultures were negative. Serial LPs on hospital days 3 and 6 remained positive for* Salmonella*. His delirium resolved on hospital day 5, and he was transferred from the MICU to a medical floor on hospital day 6. Sterilization of CSF was confirmed by negative CSF culture from LP on hospital day 10. He received 28 days of ceftriaxone once CSF sterilization was confirmed for a total of 38 days. His hospitalization was complicated by recurrent fever so further diagnostic testing was performed. Serum cryptococcal, urine* Histoplasma*, and* Blastomyces* antigen tests were obtained and were negative. On hospital day 22, mycobacterial blood cultures were positive for MAC, and his prophylactic course of azithromycin was changed to rifabutin, clarithromycin, and ethambutol for treatment of disseminated MAC. After he stabilized, a dilated ophthalmoscopic exam was normal ruling out CMV retinitis. He was ultimately discharged neurologically intact after a 44-day hospitalization.

## 3. Discussion

Meningitis is high on the differential diagnosis of any patient in the emergency department with fever and altered mental status. A large study has shown that headache (87%), neck stiffness (83%), fever (77%), and altered mental status (69%) are common presenting signs and symptoms and that, like in our case, >95% of patients diagnosed with meningitis have at least two of these four [8]. The classic clinical triad of fever, headache, and neck stiffness is present in only 44% of patients presenting with acute bacterial meningitis and is less likely to be present in nonpneumococcal meningitis.

Salmonellosis is the leading cause of foodborne infection in the developing world [4]. These infections tend to rise in HIV-endemic, developing regions where researchers estimate there are 2,000–7,500 cases of nontyphoidal salmonellosis per 100,000 HIV-infected individuals in developing countries compared to 400 cases per 100,000 HIV-infected patients in developed countries [3]. In the United States, the incidence of* Salmonella* meningitis is 0.5 to 4 cases per 100,000 adults with about 95% of* Salmonella* infections being foodborne [9].* Salmonella* meningitis remains an especially rare infection in developed regions like the United States, occurring primarily in immunocompromised adults and children under 5 years old [3].

In HIV, the depletion of CD4^+^ T-cells in the gastrointestinal tract leaves the intestinal wall prone to invasive bacterial infections by organisms like* Salmonella* Enteritidis, resulting in bacteremia and focal infections. It is known that initial HIV infection rapidly crosses the intestinal border into the lamina propria of the gut-associated lymphoid tissue (GALT). The rapid depletion of CCR5^+^ memory CD4^+^ T-cells within the GALT leaves the gut susceptible to bacterial translocation and subsequent bacteremia [10]. We believe this recession of GALT and exposure to contaminated food led to* Salmonella* bacteremia and, subsequently, meningitis.

The prevalence of* Salmonella* meningitis varies by geographic region and immunocompromised state. There are numerous cases of* Salmonella* meningitis in adult patients with previously known HIV infection living in developing countries that have been reported in the literature, but few cases of* Salmonella* meningitis in adult patients with previously known HIV infection originating in the United States have been reported.

Our literature search found only 7 reported cases of* Salmonella* meningitis in adult patients in the United States with confirmed HIV infection [1, 6, 7]. Fraimow et al. reported the first 3 cases of* Salmonella* meningitis in adults with HIV in the United States [6]. All 3 patients had group D* Salmonella* sensitive to standard antibiotics, but only 1 patient was treated successfully. Leonard et al. followed up this study with a review of 6 cases of* Salmonella* meningitis in adults with HIV in the United States, including the three reported previously [1, 6]. Of the 6 cases, 2 patients relapsed despite antibiotics and only 4 patients survived [1]. Vaughn et al. reported an adult with HIV with recurrent* Salmonella* meningitis in the United States [7].

In the literature, there is only one case report of* Salmonella* meningitis leading to the diagnosis of AIDS in a previously healthy adult [5]. In this report, a 43-year-old male in Greece presented with altered mental status and a CSF culture grew* Salmonella *Enteritidis. HIV ELISA returned positive, and CD4 count showed 16 cells/mm^3^, which confirmed AIDS. Unfortunately, the patient succumbed to refractory shock despite antibiotic therapy.

## 4. Conclusions

This is the first reported case, to our knowledge, of* Salmonella* meningitis in a previously healthy adult patient in the United States leading to a new diagnosis of AIDS.* Salmonella* meningitis has been reported in many immunocompromised adults; however, these reports describe patients with known histories of AIDS from either developing countries or HIV-endemic regions. We present this case to emphasize the importance of keeping atypical pathogens on the differential for patients with fever and altered mental status and to stress the need for evaluation of immune status as an underlying factor in meningoencephalitis caused by* Salmonella* spp.
